# Neo-Marxian social class inequalities in self-rated health among the employed in South Korea: the role of material, behavioral, psychosocial, and workplace environmental factors

**DOI:** 10.1186/s12889-017-4269-9

**Published:** 2017-04-20

**Authors:** Kyoung Ae Kong, Young-Ho Khang, Hong-Jun Cho, Sung-Mi Jang, Kyunghee Jung-Choi

**Affiliations:** 1 Department of Preventive Medicine, Ewha Womans University School of Medicine, Seoul, Korea; 2 Department of Health Policy and Management and Institute of Health Policy and Management, Seoul National University College of Medicine, Seoul, Korea; 3 Department of Family Medicine, Asan Medical Center, University of Ulsan College of Medicine, Seoul, Korea; 4 Department of Occupational and Environmental Medicine, Ewha Womans University School of Medicine, Seoul, Korea

**Keywords:** Social class, Neo-Marxist, Self-rated health, Inequality in health, Mediating factors

## Abstract

**Background:**

The aim of this study was to examine the pattern of social inequality in self-rated health among the employed using the Wright’s social class location indicator, and to assess the roles of material, behavioral, psychosocial, and workplace environmental factors as mediating factors in explaining the social class inequality in self-rated health in South Korea.

**Methods:**

This study used data from the 4th Korea National Health and Nutrition Examination Survey from 2007 to 2009. Study subjects included the employed population of 4392 men and 3309 women aged 19–64 years. Subjects were classified into twelve social class positions based on the Wright’s social class map. The health outcome was self-rated health. Material, psychosocial, behavioral, and workplace environmental factors were considered as potential mediators in explaining social class health inequality. We calculated prevalence ratios of poor self-rated health according to social class, adjusted for age and mediating factors using Poisson regression models.

**Results:**

Nonskilled workers and petty bourgeoisie reported worse self-rated health than other social classes among men. The age-adjusted prevalence of petty bourgeoisie and nonskilled workers were about four-fold greater than that of managers. Expert supervisors in the contradictory class location had a greater prevalence of poor self-rated health than experts in men. In women, the prevalence of poor self-rated health was greater in most social classes than their male counterparts, while the differences among social classes within women were not statistically significant. Workplace environmental factors explained the social class inequality by from 24 to 31% in nonskilled and skilled workers and nonskilled supervisors, respectively, and material factors showed an explanatory ability of about 8% for both nonskilled workers and petty bourgeoisie in men.

**Conclusions:**

We showed the inequality in self-rated health according to the Wright’s social class in an industrialized Asian country. Policy efforts to improve workplace environments in nonskilled and skilled workers and nonskilled supervisors would have a moderate effect on reducing the magnitude of social class inequality in self-rated health. Furthermore, the means to improve power relations in the workplace should be devised to further reduce the social class inequalities in health.

**Electronic supplementary material:**

The online version of this article (doi:10.1186/s12889-017-4269-9) contains supplementary material, which is available to authorized users.

## Background

Health inequality by socioeconomic position (SEP) is ubiquitous. Studies have reported socioeconomic health inequality using various socioeconomic position indicators [1–7]. Education, occupation, and income have been used most frequently as SEP indicators, whereas relatively few studies introduced the social class based on Neo-Marxist theory [8, 9]. However, health inequality analyses employing the Neo-Marxian approach may reveal the mechanisms of inequality through social relations of control over productive assets in capitalist societies, which cannot be captured with approaches using conventional SEP indicators [9–11].

The Wright’s class location, one of Neo-Marxian class indicators, represents power relations generating in the point of production [12]. First, those having productive assets have control over the means of production and labor force. Non-owners are exploited through the production process and it affects the distribution of income and working condition. Second, employers can delegate part of their authority to managers or supervisors. Managers and supervisors with organizational assets dominate workers using surveillance or sanctions. However, because they simultaneously belong to non-owners, they are named as a contradictory class. Third, employees are differentiated by their skill assets. Employees with high levels of skills can have the advantage within exploitation relations. In this respect, Neo-Marxian class analysis implicates the mechanism generating class inequality and can reveal different aspects of social inequality in health, which have not been explored using traditional measures of SEP [8, 9, 13].

Meanwhile, explaining the roles of the potential mediating factors between SEP and health outcome is important for policy implications. When it comes to Neo-Marxian social class, the studies exploring mediating factors have been also relatively sparse, except for a few examples [14–16]. In addition, except for a few studies using Japanese samples [17, 18], investigations into the relationship between Neo-Marxian social class and health outcomes have rarely been performed in Asian capitalist countries. South Korea, one of OECD countries, underwent rapid industrialization after the Korean War in 1950–1953, as a result of which the capitalist class and the proletariat have grown and differentiated [19, 20]. This circumstance could provide the suitable data for investigating the relationship between Neo-Marxian social class and health.

**Fig. 1:**
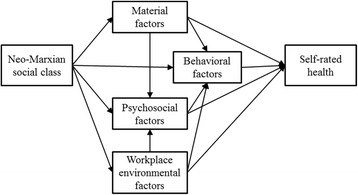
Conceptual framework for explaining social class inequality in self-rated health

A simplified conceptual framework (Fig. 1) [21] suggests four potential mediating factors explaining social class inequality in self-rated health: material [22], behavioral [23], psychosocial [22], and workplace environmental factors [14, 24]. We hypothesized that the inequality in poor self-rated health, according to Wright’s social class indicators, would be evident in South Korea. In addition, we predicted the more important roles of material and workplace environmental factors in explaining the social class inequality. Therefore, the aim of this study was to examine the pattern of socioeconomic inequality in self-rated health using Wright’s social class location indicator among the employed, and to estimate the roles of material, behavioral, psychosocial, and workplace environmental factors as mediating factors in explaining the social class inequality in self-rated health, using South Korean national samples.

## Methods

### Data and study subjects

This study was based on data obtained from the 4th Korea National Health and Nutrition Examination Survey (KNHANES), which was conducted in 2007–2009 by the Korea Centers for Disease Control and Prevention [25]. The survey used a stratified multistage probability sampling design to select about 13,800 households as a representative sample of the civilian, noninstitutionalized South Korean population. Among a total of 24,871 participants in the 4th KNHANES, this study was limited to the employed population aged 19–64 years (4392 men and 3309 women), who were employees or self-employed. Unpaid family workers were not included.

### Social class, health status, and potential mediating variables

#### Social class variables

**Fig. 2:**
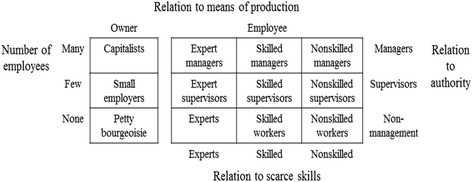
Erik Olin Wright’s class typology [12]

Subjects were classified into twelve social class positions by the three dimensions based on the Erik Olin Wright’s social class map (Fig. 2) [12], using survey questions. In the property dimension representing the relation to means of production, the respondents were classified according to whether they were self-employed. Respondents who were self-employed were classified as property owners. There were three categories of owners: capitalists, small employers, and petty bourgeoisie. Respondents who employed 10 or more workers (excluding unpaid family members) were classified as capitalists. The self-employed with 1–9 workers were classified as small employers, and those who did not hire any workers other than unpaid family member workers were classified as petty bourgeoisie.

Employees were classified into nine class positions by the combination of two dimensions - the organizational dimension (i.e., authority) and the skill dimension, each with three categories. The organizational dimension was determined by whether or not he/she had a position supervising the other workers in the workplace and by his/her occupation according to the Korean Standard Classification of Occupation (KSCO). The respondents who had a position supervising the other workers and whose occupations based on KSCO were managers were classified as managers in the organizational dimension. The respondents who had a position supervising the other workers and had occupations other than managers were classified as supervisors. Those who did not have the power of control were classified as workers.

The skill dimension was determined by occupation according to KSCO and educational level. The respondents who had completed a college education and were managers or professionals in their occupation were classified as experts. Managers or professionals with educational attainments of high school graduate or less, and clerical workers or craft and related trades workers who had completed a college education were classified as the skilled. The employees who were not assigned to the expert or skilled categories were classified as the nonskilled.

In this study, we finally used ten groups of class positions: capitalists, small employers, petty bourgeoisie, managers, expert supervisors, skilled supervisors, nonskilled supervisors, experts, skilled workers, and nonskilled workers. There was no person in the nonskilled managers group. The expert managers and the skilled managers were pooled into the managers group due to the small number of individuals.

### Health status variables

A few studies employed self-rated health as an outcome measure in the association with Neo-Marxian social class [4, 14, 26, 27]. We used the question about self-rated health status: “In general, would you say that your health is: very good, good, moderate, poor, or very poor?”. Then self-rated health was categorized into the dichotomous outcome of poor (poor and very poor) and good health status (moderate or better). Prior South Korean studies showed increased mortality risks for those who reported their health as poor or very poor while no significant mortality differences were detected among those who reported their health status as moderate, good, and very good [28, 29].

### Potential mediating variables

Material, behavioral, psychosocial, and workplace environmental factors were chosen as potential mediating variables in the association between social class and health status. Material factors were home ownership (no or one home, two or more home) and household income. Income was divided into low, middle low, middle high, and high groups using sex- and age-specific quartiles of the monthly equivalent household income.

Behavioral factors included smoking (current smokers, former smokers, and never smokers), alcohol use, and physical inactivity. Alcohol use was divided, using drinking frequency and amount over the last year, into never or nearly never drinkers (less than once per month), moderate drinkers (once per month or more, but less than the amount of high-risk drinkers), high-risk drinkers (on average, twice per week or more and on one occasion, 7 or more drinks per week in men or 5 or more drinks in women). Engagement in physical activity was defined as engaging in moderate activity for more than 30 min on 5 days per week or intense activity for more than 20 min for 3 days per week, while no engagement in physical activity was defined as having less than the level of physical activity defined above.

Psychosocial factors included the feeling of depression and the perceived level of stress. The feeling of depression was defined based on a yes or no question about the feeling of sadness or despair that lasted for at least 2 weeks during the last year. A single question with four response levels was used to ascertain perceived stress level. The answer was categorized into low (nearly none and low) and high (high and very high) groups.

Workplace environmental factors were based on the subjective interpretation on the physical workplace environment and the psychological workplace environment. The physical workplace environmental factor was obtained from four questions using the 4-point Likert scale: cleanliness of workplace, risk of workplace accidents, work in uncomfortable positions for a long time period, and workload for moving heavy loads. The psychological workplace environment was evaluated with two questions using the 4-point Likert scale: job burden and decision latitude. The four categories of the physical and psychological workplace environments were created according to the quartiles of the sum score in the corresponding questions.

### Statistical analysis

Age-standardized rates of poor self-rated health by gender were calculated by the direct method with the 2005 Korean census population being the referent. We calculated the prevalence ratio (PR) for social class adjusted for age using Poisson regression models with the robust variance [30]. As the reference category, we chose the managers class position in men considering the lowest prevalence of poor self-rated health in the group, while we used the expert supervisors in women as the reference group due to the lower poor health prevalence in the group and the insufficient number of managers.

A baseline model and additional five models were created to assess the role of each potential mediating or all potential mediating variables in explaining the association between social class and poor self-rated health. The explanatory power, that is, the degree to which the potential mediating variable explains the relationship of social class with health, was determined by the percentage change in PR, when the potential mediating variables were added to the baseline model (100 × [(PR in the baseline model) – (PR in the model adjusted for potential mediating variables)]/[(PR in the baseline model) – 1]). This method has been used to assess the contributing roles of potential mediating variables in socioeconomic inequality in health [21, 23, 31–34]. We assumed here that the roles of exposure-outcome confounders and potential mediator-outcome exposure would be minimal [35]. We presented the result of regression models only in men, because all the differences in self-rated health by social classes in women were not statistically significant. All analyses were performed using SAS statistical software (version 9.2, SAS institute, Cary, NC).

## Results

Table 1 shows the distribution of the study subjects and the prevalence of the poor self-rated health status according to social class. Among both men and women, the social class position with the largest number of subjects was nonskilled workers (24.6% in men and 41.0% in women) and the next largest social class position was petty bourgeoisie (22.3% in men and 20.7% in women). Capitalists (2.6% in men and 0.5% in women) and managers (2.7% in men and 0.4% in women) were those with the smallest number of subjects. In men, small employers, petty bourgeoisie, expert and nonskilled supervisors, and skilled and nonskilled workers had a significantly greater prevalence of poor self-rated health compared with managers. Especially, the age-adjusted prevalence of petty bourgeoisie and nonskilled workers were about four-fold greater than the prevalence of managers (PR 3.97 and 3.78, respectively). Capitalists, skilled supervisors and experts had PRs ranging between 1.85 and 2.34, but they were not statistically significant. For women, the prevalence of poor self-rated health was greater in most social classes than male counterparts, but the differences among social classes within women were not statistically significant.

**Table 1:** The distribution of study subjects and the prevalence of poor self-rated health according to Neo-Marxian social class among the employed aged 19–64 in South Korea

	Men					Women				
	No. of subjects (column %)	Poor self-rated health				No. of subjects (column %)		Poor self-rated health		
		No.	Crude rates (%)	Age-adjusted rates (%) (95% CI)	PR (95% CI)		No.	Crude rates (%)	Age-adjusted rates (%) (95% CI)	PR (95% CI)
Total	4392 (100.0)	585	13.3			3309 (100.0)	642	19.4		
Capitalists	113 (2.6)	9	8.0	5.8 (1.5–10.1)	1.85 (0.64–5.35)	17 (0.5)	5	29.4	26.5 (7.7–45.3)	2.37 (0.93–6.02)
Small employers	548 (12.5)	66	12.0	13.4 (9.6–17.2)	2.89 (1.19–7.02)	220 (6.6)	45	20.5	20.8 (14.4–27.2)	1.63 (0.86–3.12)
Petty Bourgeoisie	980 (22.3)	180	18.4	15.1 (12.1–18.2)	3.97 (1.67–9.47)	686 (20.7)	157	22.9	20.7 (17.0–24.4)	1.71 (0.93–3.15)
Managers	117 (2.7)	5	4.3	3.7 (0.5–6.9)	1.00	13 (0.4)	2	15.4	7.4 (0.0–16.0)	1.37 (0.34–5.55)
Expert supervisors	253 (5.8)	29	11.5	12.7 (7.5–18.0)	3.02 (1.20–7.62)	98 (3.0)	10	10.2	8.0 (3.3–12.8)	1.00
Skilled supervisors	375 (8.5)	33	8.8	8.6 (5.3–12.0)	2.34 (0.93–5.87)	79 (2.4)	10	12.7	7.6 (3.2–11.9)	1.26 (0.55–2.89)
Nonskilled supervisors	550 (12.35)	65	11.8	12.1 (9.2–15.1)	3.06 (1.26–7.45)	199 (6.0)	27	13.6	14.0 (9.1–18.9)	1.15 (0.58–2.30)
Expert workers	169 (3.8)	14	8.3	7.5 (3.4–11.5)	2.23 (0.82–6.04)	354 (10.7)	51	14.4	16.4 (11.0–21.8)	1.44 (0.76–2.74)
Skilled workers	206 (4.7)	20	9.7	10.7 (4.8–16.5)	2.80 (1.07–7.29)	286 (8.6)	47	16.4	25.6 (13.8–37.4)	1.76 (0.92–3.36)
Nonskilled workers	1081 (24.6)	164	15.2	14.6 (12.5–16.7)	3.78 (1.59–9.03)	1357 (41.0)	288	21.2	20.6 (18.4–22.7)	1.72 (0.94–3.13)

*CI* confidence interval, *PR* prevalence ratio

Table 2 presents the distribution of poor self-rated health by potential mediating variables in men. Low household income was related to poor self-rated health, as were feeling depressed, perceived high stress level, and poor workplace environment (Cochran-Mantel-Haenszel test, all *p* values <0.05). Never or nearly never drinkers as well as high risk drinkers were more likely to report poor health compared with moderate drinkers. Smoking was also associated with poor self-rated health, although the Cochran-Mantel-Haenszel test showed a marginal significance. The proportions of current smoker or high risk drinker were smaller in women than men, while the prevalences of women feeling depressed or with a very bad psychosocial work environment were greater in women than men. The association between potential mediating variables and poor self-rated health was all statistically significant in women (Additional file 1: Table S1).

**Table 2:** The distribution of study subjects and the prevalence of poor self-rated health according to potential mediating variables among South Korean employed men aged 19–64

			No. (column %) of subjects	No. (%) of poor self-rated health	*P* value
Total			4392 (100.0)	585 (13.3)	
Material factors	Income	Low	969 (22.1)	152 (15.7)	0.004
		Middle low	1089 (24.8)	151 (13.9)	
		Middle high	1156 (26.3)	156 (13.5)	
		High	1178 (26.8)	126 (10.7)	
	House ownership	0–1 house	3930 (89.5)	529 (13.5)	0.098
		≥ 2 houses	462 (10.5)	56 (12.1)	
Health behavioral factors	Smoking	Never	776 (17.7)	90 (11.6)	0.079
		Former	1445 (32.9)	195 (13.5)	
		Current	2171 (49.4)	300 (13.8)	
	Alcohol use	Never or nearly never drinker	929 (21.2)	150 (16.1)	0.002
		Moderate drinker	2339 (53.3)	273 (11.7)	
		High risk drinker	1124 (25.6)	162 (14.4)	
	Physical activity	No	3061 (69.7)	388 (12.7)	0.107
		Yes	1331 (30.3)	197 (14.8)	
Psychosocial factors	Feeling of depression	No	3991 (90.9)	479 (12.0)	<0.001
		Yes	401 (9.1)	106 (26.4)	
	Perceived level of stress	Nearly none	547 (12.5)	51 (9.3)	<0.001
		Low	2583 (58.8)	290 (11.2)	
		High	1043 (23.7)	180 (17.3)	
		Very high	219 (5.0)	64 (29.2)	
Workplace environmental factors	Physical environment	Very good	855 (19.5)	85 (9.9)	<0.001
		Good	1422 (32.4)	158 (11.1)	
		Bad	1303 (29.7)	186 (14.3)	
		Very bad	812 (18.5)	156 (19.2)	
	Psychological environment	Very good	663 (15.1)	78 (11.8)	<0.001
		Good	1737 (39.5)	193 (11.1)	
		Bad	1579 (36.0)	229 (14.5)	
		Very bad	413 (9.4)	85 (20.6)	

*P* values were from from Cochran-Mantel-Haenszel chi square tests with adjustment for age

Table 3 shows the distribution of potential mediating variables according to social class in men. More than 50% of capitalists belonged to the high income quartile group while over 50% of petty bourgeoisie, nonskilled supervisors, and nonskilled workers belonged to low and middle low income quartile groups. The proportion of current smokers was over 50% in capitalists, small employers, nonskilled supervisors, skilled workers and nonskilled workers. The proportion of high risk drinkers was higher in managers, capitalists, and small employers than other social class positions, while the proportion of never or nearly never drinkers was highest in expert supervisors. Capitalists and small employers showed a greater prevalence of perceived stress and feeling of depression than other social classes. Nonskilled workers, nonskilled supervisors, and petty bourgeoisie reported worse physical workplace environment than did other social classes. All types of workers as well as nonskilled supervisors had worse psychosocial workplace environment than other groups. In women, petty bourgeoisie, nonskilled supervisors, and nonskilled workers had lower income than other classes, similar to those in men (Additional file 2: Table S2). The current smoking rate was highest in nonskilled supervisors in women. Petty bourgeoisie and nonskilled workers had a greater prevalence of worse physical work environment in women (Additional file 2: Table S2).

**Table 3:** Numbers and percentages of potential mediating variables according to Neo-Marxian social class among South Korean employed men aged 19–64

			Capitalists	Small employers	Petty Bourgeoisie	Managers	Expert supervisors	Skilled supervisors	Nonskilled supervisors	Experts	Skilled workers	Nonskilled workers	*P* value
Total			113 (100.0)	548 (100.0)	980 (100.0)	117 (100.0)	253 (100.0)	375 (100.0)	550 (100.0)	169 (100.0)	206 (100.0)	1081 (100.0)	
Material factors	Income	Low	9 (8.0)	82 (15.0)	300 (30.6)	8 (6.8)	22 (8.7)	34 (9.1)	117 (21.3)	21 (12.4)	34 (16.5)	342 (31.6)	<0.001
		Middle low	19 (16.8)	125 (22.8)	264 (26.9)	19 (16.2)	36 (14.2)	85 (22.7)	160 (29.1)	26 (15.4)	50 (24.3)	305 (28.2)	
		Middle high	26 (23.0)	155 (28.3)	238 (24.3)	42 (35.9)	83 (32.8)	110 (29.3)	146 (26.5)	49 (29.0)	54 (26.2)	253 (23.4)	
		High	59 (52.2)	186 (33.9)	178 (18.2)	48 (41.0)	112 (44.3)	146 (38.9)	127 (23.1)	73 (43.2)	68 (33.0)	181 (16.7)	
	House ownership	0–1 house	97 (85.8)	461 (84.1)	883 (90.1)	101 (86.3)	226 (89.3)	326 (86.9)	501 (91.1)	146 (86.4)	188 (91.3)	1001 (92.6)	<0.001
		≥ 2 houses	16 (14.2)	87 (15.9)	97 (9.9)	16 (13.7)	27 (10.7)	49 (13.1)	49 (8.9)	23 (13.6)	18 (8.7)	80 (7.4)	
Health behavioral factors	Smoking	Never	12 (10.6)	65 (11.9)	168 (17.1)	21 (17.9)	72 (28.5)	77 (20.5)	67 (12.2)	58 (34.3)	45 (21.8)	191 (17.7)	<0.001
		Former	38 (33.6)	192 (35.0)	374 (38.2)	45 (38.5)	72 (28.5)	134 (35.7)	156 (28.4)	53 (31.4)	55 (26.7)	326 (30.2)	
		Current	63 (55.8)	291 (53.1)	438 (44.7)	51 (43.6)	109 (43.1)	164 (43.7)	327 (59.5)	58 (34.3)	106 (51.5)	564 (52.2)	
	Alcohol use	Never or nearly never	21 (18.6)	110 (20.1)	242 (24.7)	16 (13.7)	67 (26.5)	58 (15.5)	97 (17.6)	42 (24.9)	36 (17.5)	240 (22.2)	<0.001
		Moderate	61 (54.0)	277 (50.5)	498 (50.8)	68 (58.1)	126 (49.8)	224 (59.7)	302 (54.9)	103 (60.9)	125 (60.7)	555 (51.3)	
		High risk	31 (27.4)	161 (29.4)	240 (24.5)	33 (28.2)	60 (23.7)	93 (24.8)	151 (27.5)	24 (14.2)	45 (21.8)	286 (26.5)	
	Physical activity	No	84 (74.3)	384 (70.1)	665 (67.9)	89 (76.1)	196 (77.5)	293 (78.1)	364 (66.2)	130 (76.9)	159 (77.2)	697 (64.5)	<0.001
		Yes	29 (25.7)	164 (29.9)	315 (32.1)	28 (23.9)	57 (22.5)	82 (21.9)	186 (33.8)	39 (23.1)	47 (22.8)	384 (35.5)	
Psychosocial factors	Feeling of depression	No	100 (88.5)	488 (89.1)	889 (90.7)	110 (94.0)	238 (94.1)	353 (94.1)	496 (90.2)	159 (94.1)	190 (92.2)	968 (89.5)	0.042
		Yes	13 (11.5)	60 (10.9)	91 (9.3)	7 (6.0)	15 (5.9)	22 (5.9)	54 (9.8)	10 (5.9)	16 (7.8)	113 (10.5)	
	Perceived level of stress	Nearly none	7 (6.2)	71 (13.0)	170 (17.3)	10 (8.5)	26 (10.3)	26 (6.9)	48 (8.7)	25 (14.8)	18 (8.7)	146 (13.5)	<0.001
		Low	53 (46.9)	273 (49.8)	588 (60.0)	71 (60.7)	138 (54.5)	207 (55.2)	330 (60.0)	103 (60.9)	134 (65.0)	686 (63.5)	
		High	41 (36.3)	169 (30.8)	186 (19.0)	32 (27.4)	74 (29.2)	116 (30.9)	142 (25.8)	35 (20.7)	44 (21.4)	204 (18.9)	
		Very high	12 (10.6)	35 (6.4)	36 (3.7)	4 (3.4)	15 (5.9)	26 (6.9)	30 (5.5)	6 (3.6)	10 (4.9)	45 (4.2)	
Workplace environmental factors	Physical environment	Very good	36 (31.9)	109 (19.9)	110 (11.2)	43 (36.8)	104 (41.1)	137 (36.5)	73 (13.3)	76 (45.0)	50 (24.3)	117 (10.8)	<0.001
		Good	44 (38.9)	195 (35.6)	261 (26.6)	50 (42.7)	103 (40.7)	161 (42.9)	190 (34.5)	73 (43.2)	84 (40.8)	261 (24.1)	
		Bad	24 (21.2)	152 (27.7)	374 (38.2)	20 (17.1)	38 (15.0)	61 (16.3)	173 (31.5)	19 (11.2)	52 (25.2)	390 (36.1)	
		Very bad	9 (8.0)	92 (16.8)	235 (24.0)	4 (3.4)	8 (3.2)	16 (4.3)	114 (20.7)	1 (0.6)	20 (9.7)	313 (29.0)	
	Psychological environment	Very good	30 (26.5)	143 (26.1)	217 (22.1)	23 (19.7)	26 (10.3)	42 (11.2)	52 (9.5)	19 (11.2)	18 (8.7)	93 (8.6)	<0.001
		Good	44 (38.9)	227 (41.4)	388 (39.6)	68 (58.1)	118 (46.6)	167 (44.5)	225 (40.9)	59 (34.9)	73 (35.4)	368 (34.0)	
		Bad	35 (31.0)	159 (29.0)	324 (33.1)	24 (20.5)	85 (33.6)	140 (37.3)	226 (41.1)	67 (39.6)	83 (40.3)	436 (40.3)	
		Very bad	4 (3.5)	19 (3.5)	51 (5.2)	2 (1.7)	24 (9.5)	26 (6.9)	47 (8.5)	24 (14.2)	32 (15.5)	184 (17.0)	

Table 4 shows the explanatory power of potential mediating variables in explaining the association between social classes and poor self-rated health. In models 2 to 5 including each potential mediating variable, the explanatory power of workplace environmental factors was highest in all social class positions except capitalists. The PR change due to workplace environmental factors in model 5 was about 31% in nonskilled workers and about 24% in skilled workers and nonskilled supervisors. Workplace environmental factors explained excess prevalence ratios in other classes by 11–19%. The PR reduction with adjustment of material, behavioral, and psychosocial factors mostly recorded less than 10%. Material factors in model 2 explained the excess prevalence ratio in nonskilled workers by 8.1% and in petty bourgeoisie by 7.8%. Behavioral factors in model 3 showed explanatory powers of about 5% in explaining the excess prevalence ratio in small employers, petty bourgeoisie, nonskilled supervisors and nonskilled workers. Introduction of psychosocial factors into model 4 lowered PR of small employers by 9.6%. The adjustment of all potential mediating variables in model 6 showed an explanatory power of about 25% in nonskilled workers, nonskilled supervisors and small employers and only about 10% in expert supervisors.

**Table 4:** The explanatory power of potential mediating variables in explaining the relationship between social class and poor self-rated health among South Korean employed men aged 19–64. (reference group: managers)

		Capitalists	Small employers	Petty Bourgeoisie	Expert supervisors	Skilled supervisors	Nonskilled supervisors	Experts	Skilled workers	Nonskilled workers
Model 1: Baseline model	PR (95% CI)	1.85 (0.64–5.35)	2.89 (1.19–7.02)	3.97 (1.67–9.47)	3.02 (1.20–7.62)	2.34 (0.93–5.87)	3.06 (1.26–7.45)	2.23 (0.82–6.04)	2.80 (1.07–7.29)	3.78 (1.59–9.03)
Model 2: Baseline + material factors	PR (95% CI)	1.88 (0.65–5.43)	2.84 (1.17–6.91)	3.74 (1.57–8.93)	3.04 (1.20–7.66)	2.34 (0.93–5.87)	2.94 (1.21–7.17)	2.23 (0.83–6.05)	2.75 (1.05–7.16)	3.56 (1.49–8.51)
	PR change (%)	−3.3	2.7	7.8	−0.9	0.3	5.8	−0.2	2.8	8.1
Model 3: Baseline + behavioral factors	PR (95% CI)	1.79 (0.62–5.17)	2.78 (1.15–6.76)	3.83 (1.61–9.11)	2.98 (1.18–7.49)	2.38 (0.95–5.95)	2.95 (1.21–7.17)	2.30 (0.85–6.21)	2.81 (1.08–7.30)	3.63 (1.52–8.66)
	PR change (%)	7.3	5.7	4.9	2.0	−2.5	5.6	−5.4	−0.4	5.6
Model 4: Baseline + psychosocial factors	PR (95% CI)	1.55 (0.54–4.46)	2.71 (1.13–6.50)	4.08 (1.73–9.58)	2.99 (1.21–7.42)	2.25 (0.91–5.57)	3.01 (1.26–7.23)	2.39 (0.90–6.39)	2.87 (1.12–7.38)	3.88 (1.65–9.13)
	PR change (%)	35.2	9.6	−3.5	1.3	6.7	2.4	−13.1	−4.2	−3.5
Model 5: Baseline + workplace environmental factors	PR (95% CI)	1.73 (0.60–5.01)	2.61 (1.07–6.35)	3.40 (1.42–8.15)	2.80 (1.11–7.05)	2.17 (0.86–5.43)	2.57 (1.05–6.26)	2.03 (0.75–5.49)	2.35 (0.90–6.15)	2.92 (1.21–7.01)
	PR change (%)	13.8	15.0	19.2	11.1	13.1	24.1	16.8	24.8	31.1
Model 6: Baseline + all factors	PR (95% CI)	1.48 (0.52–4.24)	2.46 (1.03–5.87)	3.45 (1.47–8.10)	2.81 (1.14–6.94)	2.16 (0.88–5.33)	2.56 (1.06–6.14)	2.29 (0.86–6.11)	2.56 (1.00–6.56)	3.04 (1.29–7.20)
	PR change (%)	43.3	23.0	17.7	10.5	13.3	24.5	−4.6	13.5	26.6

*CI* confidence interval, *PR* prevalence ratio. PR change: percentage change in prevalence ratio = 100×((PR in baseline model) – (PR in model adjusted for risk factor))/((PR in baseline model) – 1). The baseline model (model 1) for prevalence ratio refers to the model only adjusted for age

## Discussion

This study showed the inequality in poor self-rated health according to the Wright’s social class in men. The risk of poor self-rated health was highest among petty bourgeoisie and nonskilled workers, which was modestly accounted for by workplace environmental factors and material factors.

The poor health status of petty bourgeoisie found in this study was somewhat different from the finding in a study of the city of Barcelona in which petty bourgeoisie’s self-rated health was better than the supervisors [14]. The subjects of this study included individuals from rural areas as well as cities in South Korea. Petty bourgeoisie from rural areas accounted for 43.9% of total male petty bourgeoisie in this study. Our additional analyses showed that the indirectly standardized prevalence of poor self-rated health among rural petty bourgeoisie was 20.1% while the prevalence among urban petty bourgeoisie was 17.0%. This result was similar to that of a previous study in which the self-employed in the agricultural sector reported much worse self-rated health than those in other social strata [36]. Korean economic development has been accomplished by focusing on industrialization and urbanization since the 1960s [37]. In this process, socioeconomic conditions in rural areas have worsened, and a higher mortality rate in rural areas than urban areas was reported [38].

The other reason of the worst self-rated health of petty bourgeoisie could be derived from the characteristic of the class itself and the change of its environment for survival. Petty bourgeoisie are free from the exploitation, however, at the same time, must use their own labor for an income unlike rentiers [12, 39]. Given the increasing infiltration of capitals in conglomerates into the sectors of retail and food service in Korea, the economic environment has been more competitive for petty bourgeoisie than in the past [20]. It turned into their reducing income [20], and it could prevent the investment for the improvement of physical working environment and develop worse psychosocial working environment. In view of this, the result of this study that the workplace environmental factor was important for explaining the worse health status of petty bourgeoisie could be understood, and that was also consistent with a previous study [14].

Small employers had a worse perceived health status than managers, which was consistent with previous studies [4, 14]. The intensified polarization between conglomerates and small-sized enterprises along with the high risk of bankruptcy in small-sized enterprises has been reported since the 1990s in Korea [40, 41]. The uncertain future of an owned small enterprise might have created an unfavorable perception about health among small employers. In a previous European study, small employers had a higher level of stress than employees [42]. Our analysis results also showed that the proportion of small employers with high level of perceived stress were relatively high (see Table 3).

The worst health status of nonskilled workers has been reported in other studies from Western countries [14, 15, 43–45]. This study adds an additional support for these prior findings in a relatively newly developed Asian capitalist country. The extent of explanation for the increased risk of poor health by workplace environmental factor (31%) and material factor (8%) was highest in nonskilled workers among social classes (see Table 4). As shown in Table 3, the ‘very bad’ physical and psychosocial working environments were concentrated on nonskilled workers who did not have any property, organizational, and skill assets. Moreover, almost 60% of nonskilled workers had a lower then median income in our data (Table 3). These findings suggest that nonskilled workers in South Korea were sharply experiencing exploitation through low income and uncontrollable working environment [9, 43].

Supervisors are one of the contradictory class positions [12, 46, 47]. The exploitation form capitalists with the insufficient authority over workers places supervisors between managers and workers and may subsequently impose worse psychological burden on them. Their general and mental health status was reportedly worse than experts or skilled workers in previous studies [14, 47]. Our study also provided evidence that expert supervisors and nonskilled supervisors might have worse self-rated health than expert workers and skilled workers as well as managers.

The study results suggest that the different level of skill assets seem to have different effects on perceived health among supervisors. The self-rated health of non-skilled supervisors tends to be better than that of workers with the same level of skill (nonskilled workers). Analysis results indicated that the psychosocial and physical working environment and income status of nonskilled supervisors tended to be better than nonskilled workers but worse than expert workers and skilled workers as well as other supervisors. Among nonskilled supervisors, relatively low organizational assets might have positively affected on perceived health through working environment and income, despite the adverse health impact of low skill assets. On the other hand, the prevalence of poor self-rated health of expert supervisors tended to be higher than expert workers with the same levels of skill. In other words, the social group with relatively low level organizational assets and high level skill assets was more likely to have worse perceived health status than the social group with no organizational assets and high level skill assets. Prior studies showed inconsistent findings on the relative health advantage of expert supervisors compared with other social groups [4, 15]. A Spanish study showed that expert supervisors had better self-rated health and suggested that the skill assets might have played a crucial role in health status [4]. However, in a European study, expert supervisors reported worse mental well-being than non-expert supervisors, which was explained by the concept of ‘status incongruence’ [15, 48]. Expert supervisors could be considered as having high status congruence, because they had high skill assets and, at the same time, organizational assets. However, expert supervisors, as contradictory class location, could be controlled from the owner of productive assets, which might lead poor mental health. This concept of ‘status incongruence’ might be partly applied to our results. Future studies need to explore the interaction between organizational assets and skill assets and their effect on health.

Prior studies showed that the worst health status was found in nonskilled workers among social classes in women [4, 14, 15]. We also analyzed the differences in self-rated health by social class in women, but could not show a significantly increased risk of poor health among nonskilled female workers. The small sample size might have contributed to the finding. Furthermore, the labor market situation for South Korean women might have played a role in creating the weak social class inequality in poor self-rated health. A prior comparative study between Britain and Finland suggested the possibility that the women in a society with low employment participation rate and high rate of part time work might show a weak effect of their own occupational class and a strong effect of their household roles on health status [49]. In Korea, the average labor force participation rate of women aged 15–64 was only 54.7% [50] and about 70% of women in the labor force were in nonstandard jobs in 2008 [51]. In addition, the average domestic work time of married and employed women was over 10 times greater than married and employed men [52], which might have created a ‘dual burden’ on employed women, affected women’s poor self-rated health [14, 53, 54], and subsequently attenuated the magnitude of the effect of occupational class. It should be noted that, in most social classes, the self-rated health in women was poorer than that in women (Table 1). The pattern and mechanism of social inequality in health in South Korean women should be explored, considering both women’s labor environment at workplace and power relations at homes.

Our findings should be interpreted in light of several limitations. First, we used a cross-sectional sample, so could not draw the causal direction of the relationships. Second, due to the restriction of secondary data, this study used only one point value of social class and potential mediating variables. This might have prevented from sufficiently revealing roles of Neo-Marxian social class on health and might have contributed to the relatively weak explanatory power of the potential mediating factors. Third, we could not have the sufficient sample size within each social class of twelve, so managers were analyzed as one group without differentiation of credential assets. However, this study showed social class inequality in self-rated health in a newly industrialized non-Western capitalist country with nationally representative employed population including both employees and self-employed, which may contribute to the discussion about the socioeconomic inequality in health with the Neo-Marxist approach.

## Conclusions

We identified the inequality in self-rated health according to the Wright’s social class articulated by the ownership of the means of production, organizational assets, and skill assets at the point of production in an industrialized Asian country. The risk of poor self-rated health was highest among petty bourgeoisie and nonskilled workers, which was modestly accounted for by workplace environmental factors and material factors. Policy efforts to improve workplace environment in nonskilled and skilled workers and nonskilled supervisors would have a moderate effect on reducing the magnitude of social class inequality in self-rated health. Furthermore, the means to improve power relations in the workplace should be devised to further reduce the social class inequalities in health.

## Additional files

Additional file 1: Table S1. The distribution of study subjects and the prevalence of poor-self-rated health according to potential mediating variables among South Korean employed women aged 19–64. (DOCX 40 kb)

Additional file 2: Table S2. Numbers and percentages of potential mediating variables according to Neo-Marxian social class among South Korean employed women aged 19–64. (DOCX 45 kb)

## Abbreviations

KNHANES: Korea National Health and Nutrition Examination Survey
KSCO: Korean Standard Classification of Occupation
PR: Prevalence ratio
SEP: Socioeconomic position
