# Supplementary material for: Neo-Marxian social class inequalities in self-rated health among the employed in South Korea: the role of material, behavioral, psychosocial, and workplace environmental factors
Source: BMC Public Health. 2017 Apr 20;17:345. doi: 10.1186/s12889-017-4269-9 (PMC5397726; doi:10.1186/s12889-017-4269-9)
Supplement: Supplementary file 1 — The distribution of study subjects and the prevalence of poor-self-rated health according to potential mediating variables among South Korean employed women aged 19–64. (DOCX 40 kb) [file 12889_2017_4269_MOESM1_ESM.docx]

Supplemental table 1. The distribution of study subjects and the prevalence of poor-self-rated health according to potential mediating variables among South Korean employed women aged 19-64.

|  |  |  | No. (column %) of subjects | No. (%) of poor self-rated health | P value |
| --- | --- | --- | --- | --- | --- |
| Total |  |  | 3309 (100.0) | 642 (19.4) |  |
| Material factors | Income | Low | 826 (25.0) | 192 (23.2) | 0.003 |
|  |  | Middle low | 806 (24.4) | 169 (21.0) |  |
|  |  | Middle high | 819 (24.8) | 137 (16.7) |  |
|  |  | High | 858 (25.9) | 144 (16.8) |  |
|  | House ownership | 0–1 house | 2969 (89.7) | 588 (19.8) | 0.002 |
|  |  | ≥ 2 houses | 340 (10.3) | 54 (15.9) |  |
| Health behavioral factors | Smoking | Never | 2905 (87.8) | 539 (18.6) | <0.001 |
|  |  | Former | 159 (4.8) | 32 (20.1) |  |
|  |  | Current | 245 (7.4) | 71 (29.0) |  |
|  | Alcohol use | Never or nearly never drinker | 1647 (49.8) | 338 (20.5) | 0.021 |
|  |  | Moderate drinker | 1433 (43.3) | 249 (17.4) |  |
|  |  | High risk drinker | 229 (6.9) | 55 (24.0) |  |
|  | Physical activity | No | 2450 (74.0) | 446 (18.2) | 0.013 |
|  |  | Yes | 859 (26.0) | 196 (22.8) |  |
| Psychosocial factors | Feeling of depression | No | 2734 (82.6) | 462 (16.9) | <0.001 |
|  |  | Yes | 575 (17.4) | 180 (31.3) |  |
|  | Perceived level of stress | Nearly none | 332 (10.0) | 47 (14.2) | <0.001 |
|  |  | Low | 1862 (56.3) | 274 (14.7) |  |
|  |  | High | 934 (28.2) | 238 (25.5) |  |
|  |  | Very high | 181 (5.5) | 83 (45.9) |  |
| Workplace environmental factors | Physical environment | Very good | 798 (24.1) | 115 (14.4) | <0.001 |
|  |  | Good | 1275 (38.5) | 199 (15.6) |  |
|  |  | Bad | 909 (27.5) | 219 (24.1) |  |
|  |  | Very bad | 327 (9.9) | 109 (33.3) |  |
|  | Psychological environment | Very good | 402 (12.1) | 55 (13.7) | <0.001 |
|  |  | Good | 1164 (35.2) | 178 (15.3) |  |
|  |  | Bad | 1221 (36.9) | 258 (21.1) |  |
|  |  | Very bad | 522 (15.8) | 151 (28.9) |  |

P values were from Cochran-Mantel-Haenszel chi square tests with adjustment for age.
