# Supplementary material for: Neo-Marxian social class inequalities in self-rated health among the employed in South Korea: the role of material, behavioral, psychosocial, and workplace environmental factors
Source: BMC Public Health. 2017 Apr 20;17:345. doi: 10.1186/s12889-017-4269-9 (PMC5397726; doi:10.1186/s12889-017-4269-9)
Supplement: Supplementary file 2 — Numbers and percentages of potential mediating variables according to Neo-Marxian social class among South Korean employed women aged 19–64. (DOCX 45 kb) [file 12889_2017_4269_MOESM2_ESM.docx]

Supplemental table 2. Numbers and percentages of potential mediating variables according to Neo-Marxian social class among South Korean employed women aged 19-64.

|  |  |  | Capitalists | Small employers | Petty Bourgeoisie | Managers | Expert Supervisors | Skilled Supervisors | Nonskilled supervisors | Experts | Skilled workers | Nonskilled workers |
| --- | --- | --- | --- | --- | --- | --- | --- | --- | --- | --- | --- | --- |
| Total |  |  | 17 (100.0) | 220 (100.0) | 686 (100.0) | 13 (100.0) | 98 (100.0) | 79 (100.0) | 199 (100.0) | 354 (100.0) | 286 (100.0) | 1357 (100.0) |
| Material factors | Income | Low | 0 (0.0) | 30 (13.6) | 188 (27.4) | 0 (0.0) | 19 (19.4) | 9 (11.4) | 47 (23.6) | 47 (13.3) | 56 (19.6) | 430 (31.7) |
|  |  | Middle low | 1 (5.9) | 41 (18.6) | 169 (24.6) | 2 (15.4) | 15 (15.3) | 6 (7.6) | 50 (25.1) | 61 (17.2) | 59 (20.6) | 402 (29.6) |
|  |  | Middle High | 4 (23.5) | 61 (27.7) | 170 (24.8) | 6 (46.2) | 27 (27.6) | 24 (30.4) | 45 (22.6) | 82 (23.2) | 72 (25.2) | 328 (24.2) |
|  |  | High | 12 (70.6) | 88 (40.0) | 159 (23.2) | 5 (38.5) | 37 (37.8) | 40 (50.6) | 57 (28.6) | 164 (46.3) | 99 (34.6) | 197 (14.5) |
|  | House ownership | 0–1 house | 15 (88.2) | 180 (81.8) | 599 (87.3) | 10 (76.9) | 80 (81.6) | 73 (92.4) | 180 (90.5) | 312 (88.1) | 249 (87.1) | 1271 (93.7) |
|  |  | ≥ 2 houses | 2 (11.8) | 40 (18.2) | 87 (12.7) | 3 (23.1) | 18 (18.4) | 6 (7.6) | 19 (9.5) | 42 (11.9) | 37 (12.9) | 86 (6.3) |
| Health behavioral factors | Smoking | Never | 15 (88.2) | 180 (81.8) | 616 (89.8) | 11 (84.6) | 94 (95.9) | 69 (87.3) | 162 (81.4) | 334 (94.4) | 257 (89.9) | 1167 (86.0) |
|  |  | Former | 1 (5.9) | 20 (9.1) | 28 (4.1) | 1 (7.7) | 2 (2.0) | 6 (7.6) | 12 (6.0) | 11 (3.1) | 14 (4.9) | 64 (4.7) |
|  |  | Current | 1 (5.9) | 20 (9.1) | 42 (6.1) | 1 (7.7) | 2 (2.0) | 4 (5.1) | 25 (12.6) | 9 (2.5) | 15 (5.2) | 126 (9.3) |
|  | Alcohol use | Never or nearly never | 6 (35.3) | 97 (44.1) | 385 (56.1) | 8 (61.5) | 55 (56.1) | 36 (45.6) | 83 (41.7) | 198 (55.9) | 144 (50.3) | 635 (46.8) |
|  |  | Moderate | 9 (52.9) | 101 (45.9) | 254 (37.0) | 5 (38.5) | 41 (41.8) | 39 (49.4) | 94 (47.2) | 143 (40.4) | 134 (46.9) | 613 (45.2) |
|  |  | High risk | 2 (11.8) | 22 (10.0) | 47 (6.9) | 0 (0.0) | 2 (2.0) | 4 (5.1) | 22 (11.1) | 13 (3.7) | 8 (2.8) | 109 (8.0) |
|  | Physical activity | No | 11 (64.7) | 156 (70.9) | 499 (72.7) | 10 (76.9) | 70 (71.4) | 63 (79.7) | 147 (73.9) | 300 (84.7) | 235 (82.2) | 959 (70.7) |
|  |  | Yes | 6 (35.3) | 64 (29.1) | 187 (27.3) | 3 (23.1) | 28 (28.6) | 16 (20.3) | 52 (26.1) | 54 (15.3) | 51 (17.8) | 398 (29.3) |
| Psychosocial factors | Feeling of depression | No | 13 (76.5) | 180 (81.8) | 549 (80.0) | 10 (76.9) | 84 (85.7) | 65 (82.3) | 161 (80.9) | 305 (86.2) | 252 (88.1) | 1115 (82.2) |
|  |  | Yes | 4 (23.5) | 40 (18.2) | 137 (20.0) | 3 (23.1) | 14 (14.3) | 14 (17.7) | 38 (19.1) | 49 (13.8) | 34 (11.9) | 242 (17.8) |
|  | Perceived level of stress | Nearly none | 0 (0.0) | 15 (6.8) | 79 (11.5) | 0 (0.0) | 6 (6.1) | 3 (3.8) | 22 (11.1) | 38 (10.7) | 29 (10.1) | 140 (10.3) |
|  |  | Low | 8 (47.1) | 120 (54.5) | 409 (59.6) | 8 (61.5) | 44 (44.9) | 47 (59.5) | 104 (52.3) | 190 (53.7) | 158 (55.2) | 774 (57.0) |
|  |  | High | 7 (41.2) | 76 (34.5) | 154 (22.4) | 4 (30.8) | 39 (39.8) | 25 (31.6) | 62 (31.2) | 107 (30.2) | 87 (30.4) | 373 (27.5) |
|  |  | Very high | 2 (11.8) | 9 (4.1) | 44 (6.4) | 1 (7.7) | 9 (9.2) | 4 (5.1) | 11 (5.5) | 19 (5.4) | 12 (4.2) | 70 (5.2) |
| Workplace environmental factors | Physical environment | Very good | 4 (23.5) | 47 (21.4) | 148 (21.6) | 5 (38.5) | 35 (35.7) | 36 (45.6) | 35 (17.6) | 112 (31.6) | 129 (45.1) | 247 (18.2) |
|  |  | Good | 7 (41.2) | 78 (35.5) | 249 (36.3) | 4 (30.8) | 39 (39.8) | 32 (40.5) | 90 (45.2) | 172 (48.6) | 107 (37.4) | 497 (36.6) |
|  |  | Bad | 3 (17.6) | 74 (33.6) | 207 (30.2) | 4 (30.8) | 21 (21.4) | 11 (13.9) | 57 (28.6) | 58 (16.4) | 43 (15.0) | 431 (31.8) |
|  |  | Very bad | 3 (17.6) | 21 (9.5) | 82 (12.0) | 0 (0.0) | 3 (3.1) | 0 (0.0) | 17 (8.5) | 12 (3.4) | 7 (2.4) | 182 (13.4) |
|  | Psychological environment | Very good | 0 (0.0) | 39 (17.7) | 137 (20.0) | 2 (15.4) | 10 (10.2) | 7 (8.9) | 19 (9.5) | 36 (10.2) | 28 (9.8) | 124 (9.1) |
|  |  | Good | 7 (41.2) | 82 (37.3) | 251 (36.6) | 2 (15.4) | 30 (30.6) | 29 (36.7) | 90 (45.2) | 135 (38.1) | 120 (42.0) | 418 (30.8) |
|  |  | Bad | 8 (47.1) | 85 (38.6) | 245 (35.7) | 9 (69.2) | 47 (48.0) | 35 (44.3) | 61 (30.7) | 133 (37.6) | 95 (33.2) | 503 (37.1) |
|  |  | Very bad | 2 (11.8) | 14 (6.4) | 53 (7.7) | 0 (0.0) | 11 (11.2) | 8 (10.1) | 29 (14.6) | 50 (14.1) | 43 (15.0) | 312 (23.0) |
